# Human Clinical Isolates of Pathogenic Fungi Are Host to Diverse Mycoviruses

**DOI:** 10.1128/spectrum.01610-22

**Published:** 2022-08-22

**Authors:** Cormac M. Kinsella, Martin Deijs, H. M. Gittelbauer, Lia van der Hoek, Karin van Dijk

**Affiliations:** a Amsterdam UMC, Laboratory of Experimental Virology, Department of Medical Microbiology and Infection Prevention, University of Amsterdam, Amsterdam, The Netherlands; b Amsterdam Institute for Infection and Immunity, Amsterdam, The Netherlands; c Amsterdam UMC, Laboratory of Mycology, Department of Medical Microbiology and Infection Prevention, University of Amsterdam, Amsterdam, The Netherlands; University of California, San Diego

**Keywords:** virus discovery, fungi, human clinical isolate, mycovirus, jivivirus, contamination, jivivirus host

## Abstract

Fungi host viruses from many families, and next-generation sequencing can be used to discover previously unknown genomes. Some fungus-infecting viruses (mycoviruses) confer hypovirulence on their pathogenic hosts, raising the possibility of therapeutic application in the treatment of fungal diseases. Though all fungi probably host mycoviruses, many human pathogens have none documented, implying the mycoviral catalogue remains at an early stage. Here, we carried out virus discovery on 61 cultures of pathogenic fungi covering 27 genera and at least 56 species. Using next-generation sequencing of total nucleic acids, we found no DNA viruses but did find a surprising RNA virus diversity of 11 genomes from six classified families and two unclassified lineages, including eight genomes likely representing new species. Among these was the first jivivirus detected in a fungal host (Aspergillus lentulus). We separately utilized rolling circle amplification and next-generation sequencing to identify ssDNA viruses specifically. We identified 13 new cressdnaviruses across all libraries, but unlike the RNA viruses, they could not be confirmed by PCR in either the original unamplified samples or freshly amplified nucleic acids. Their distributions among sequencing libraries and inconsistent detection suggest low-level contamination of reagents. This highlights both the importance of validation assays and the risks of viral host prediction on the basis of highly amplified sequencing libraries. Meanwhile, the detected RNA viruses provide a basis for experimentation to characterize possible hypovirulent effects, and hint at a wealth of uncharted viral diversity currently frozen in biobanks.

**IMPORTANCE** Fungal pathogens of humans are a growing global health burden. Viruses of fungi may represent future therapeutic tools, but for many fungal pathogens there are no known viruses. Our study examined the viral content of diverse human-pathogenic fungi in a clinical biobank, identifying numerous viral genomes, including one lineage previously not known to infect fungi.

## INTRODUCTION

The risk of life-threatening invasive fungal infections (IFIs) has been growing for decades (1, 2), partly due to use of immunosuppressive drugs and chemotherapy, though the cause is thought to be multifactorial (3). A shifting epidemiology has also been observed, with once-rare pathogens becoming significant concerns (4). While historically, *Candida* yeasts and *Aspergillus* moulds have caused the majority of IFIs in cancer patients and hematopoietic stem cell transplant recipients, recently *Rhizopus*, *Mucor*, *Fusarium*, and others have emerged as threats (3–5). Next to that, disseminated infections with dimorphic environmental fungi such as Histoplasma capsulatum and Blastomyces dermatitidis have been described in neonates and immunocompromised patients within regions of endemicity (6). Meningoencephalitis due to Cryptococcus neoformans and Cryptococcus gattii is often seen in HIV-positive patients (7), while the recent COVID-19 pandemic has had a tangible impact, with COVID-19-associated pulmonary aspergillosis described in around 30% of ICU patients (8, 9). In Italy and Brazil, up to a 10-fold increase in candidemia has been reported in patients with COVID-19 (10, 11), and in India, a high incidence of mucormycosis is found in COVID-19 patients, with a mortality rate of 35% (12).

Fungal infections are difficult to treat, with only a few options available and resistance to these emerging (e.g., azole resistance in invasive Aspergillus fumigatus), while emerging species may be unaffected by standard empirical treatments (3). Available antifungal drugs also have marked side effects, like nephrotoxicity and hepatotoxicity. New treatments that can combat fungal infections would be valuable. Like any living creature, fungi are susceptible to viral infection. Viruses of fungi are called mycoviruses, though this term encompasses a massive genetic diversity spanning viruses of many lineages (13). Known mycoviruses almost all have RNA genomes, though recently three ssDNA mycoviruses of the family *Genomoviridae* were identified (14–16), and endogenous viral elements found in fungal genomes hint that other species of this family also infect fungi (17). If infection by a virus slows or halts growth of a fungal pathogen, for example via cell lysis, this can cause reduced virulence during infection (hypovirulence). Hypovirulence-associated viruses may provide future options for antifungal therapies, and mycoviruses are in fact already applied in the biological control of fungal phytopathogens. An example is chestnut blight, a disease of chestnut trees caused by the fungus *Cryphonectria parasitica*. Infected trees can be treated with the RNA virus Cryphonectria hypovirus 1 (CHV1), resulting in a significantly reduced virulence of the fungus (18, 19). Another example is treatment of Sclerotinia sclerotiorum infection of plants using the virus Sclerotinia sclerotiorum hypovirulence-associated DNA virus 1 (SsHADV-1) (14). Infecting the fungus on rapeseed plants reduces disease severity and enhances the rapeseed yield (20). Another mycovirus treatment under development for plants is Rosellinia necatrix megabirnavirus 1 (RnMBV1), which infects the fungal species causing white root rot of fruit trees (21).

Not all mycoviruses are viable as biological control agents, especially for fungal pathogens of humans. It may be important that the virus is not recognized by the innate immune system (e.g., Toll-like receptors [22]) or the adaptive immune system, and thus a low viral antigenicity is preferable. Viruses should be deliverable to a target fungus in the patient using application techniques such as injection or topical administration. For this, an extracellular phase would be ideal, but the majority of known mycoviruses lack one (23, 24). Without an extracellular phase, possible options for virus infection are more complex; for example, hyphal anastomosis between the patient strain and a virus-infected conspecific strain would require addition of a hypovirulent fungus (e.g., infected conidia), which is unlikely a viable approach in human patients. Notably, SsHADV-1 was both the first known DNA mycovirus and the first mycovirus confirmed to have an extracellular phase (24). This raises the possibility that ssDNA viruses represent the likeliest candidates for therapeutic applications in humans (25). In order to identify unknown mycoviruses that may have utility in future therapeutics, we investigated cultured clinical isolates of human-pathogenic fungi, using virus discovery cDNA-AFLP (VIDISCA) next-generation sequencing and Illumina sequencing with or without initial rolling circle amplification.

## RESULTS

### RNA virus discovery in clinical isolates.

**FIG 1:**
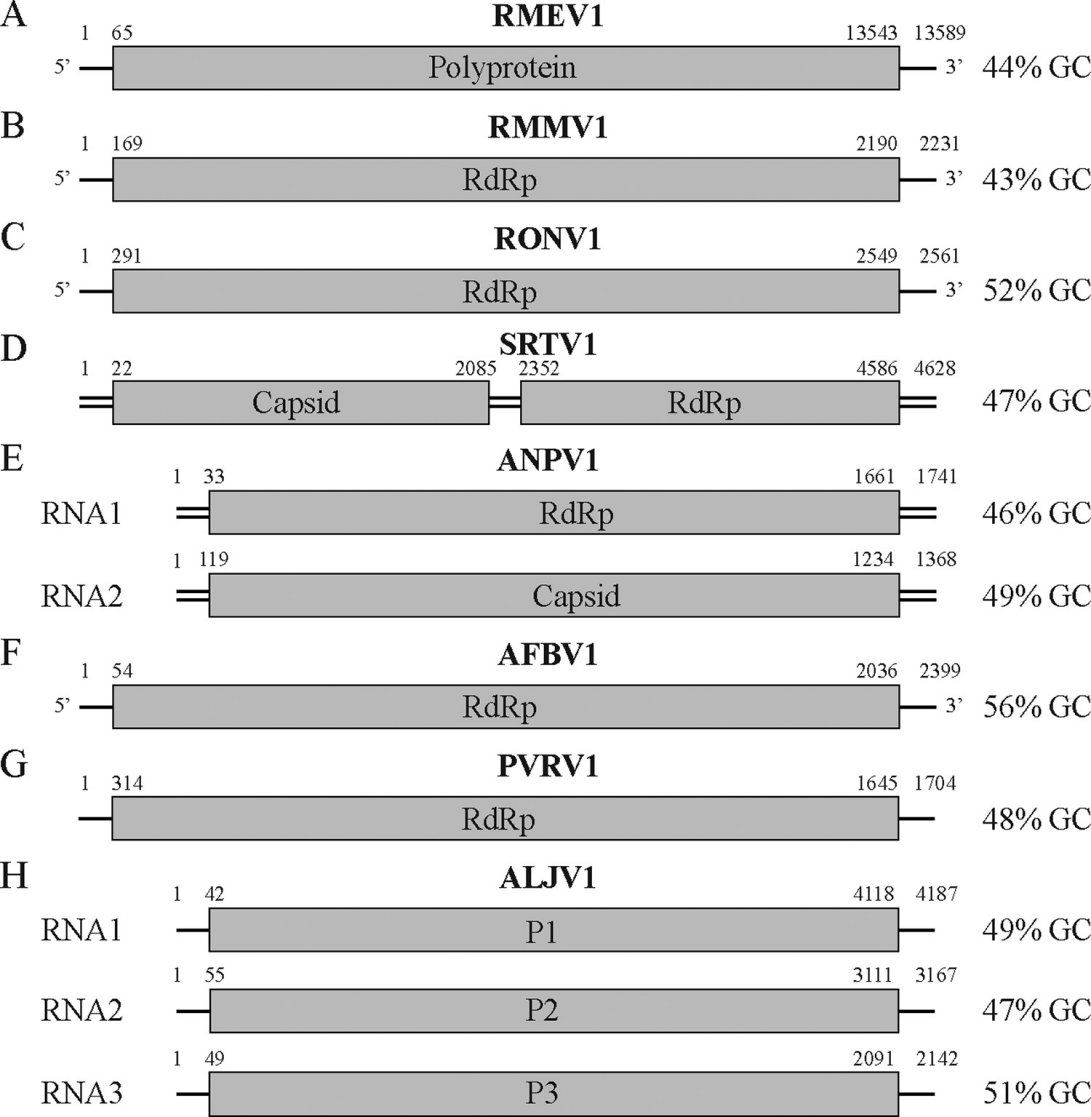
Genome organization of representative RNA viruses for each identified taxonomic group. (A) *Endornaviridae*, Rhizopus microsporus endornavirus 1 (RMEV1, LC671616). (B) *Mitoviridae*, Rhizopus microsporus mitovirus 1 (RMMV1, LC671615). (C) *Narnaviridae*, Rhizopus oryzae narnavirus 1 (RONV1, LC671613). (D) *Totiviridae*, Syncephalastrum racemosum totivirus 1 (SRTV1, LC671614). (E) *Partitiviridae*, Aspergillus niger partitivirus 1 (ANPV1, LC671611 and LC671612). (F) *Botourmiaviridae*, Aspergillus fumigatus botourmiavirus 1 (AFBV1, LC671624). (G) Unclassified, Penicillium vanoranjei associated RNA virus 1 (PVRV1, LC671619). (H) Unclassified, Aspergillus lentulus jivivirus 1 (ALJV1, LC671620, LC671621, and LC671622). Viral sense is not shown for dsRNA viruses and those with unknown sense. Genome sizes are not drawn to scale.

The 61 initial fungal samples belonged to 27 genera and at least 56 species (Table S1 in the supplemental material). VIDISCA sequencing produced an average of 7,600 reads per sample. Bioinformatic analysis identified six samples containing between 1 and 1,306 RNA virus reads belonging to distinct viral lineages. To recover and characterize full genomes of these viruses, Illumina reads were generated (an average of 3.5 million reads per sample after quality control) and assembled from the six positive samples plus one without detected viruses to serve as a control of screening sensitivity. Sequenced isolates belonged to Rhizopus microsporus, R. oryzae, Syncephalastrum racemosum, Aspergillus niger, A. lentulus, Cladosporium sphaerospermum, and Penicillium vanoranjei. Surprisingly, upon analysis we found that all resulting assemblies, including the control, contained at least one RNA virus, and two had mixed infections with three viral species each (Table 1). A total of 11 RNA viruses were therefore found instead of the six expected. BLASTx searches showed these belonged to the six families *Partitiviridae*, *Narnaviridae*, *Totiviridae*, *Mitoviridae*, *Endornaviridae*, and *Botourmiaviridae*, plus two unclassified lineages: a jivivirus related to the family *Virgaviridae* and a ribovirus with uncertain relationships (Fig. 1). PCR screening confirmed the presence of all RNA viruses in their respective index samples, and even detected additional positive samples for four of the viruses (Table S3). Three of these additional detections were made in fungi belonging to the same genus as the index (*Rhizopus*, *Aspergillus*, and *Rhizomucor*), but a different species. The last was made in a different genus (*Trichophyton*) to the index (*Aspergillus*), both of which are in the class *Eurotiomycetes*.

**TABLE 1:** RNA viruses metagenomically sequenced from clinical isolates of fungi

Sample	Host	Virus family	Virus genus	Putative viral genome	Molecule	Accession
4	A. niger	*Partitiviridae*	*Gammapartitivirus*	Aspergillus niger partitivirus 1	dsRNA	LC671611 LC671612
11	R. oryzae	*Narnaviridae*	Unclassified	Rhizopus oryzae narnavirus 1	ssRNA(+)	LC671613
13	S. racemosum	*Totiviridae*	*Totivirus*	Syncephalastrum racemosum totivirus 1	dsRNA	LC671614
15	R. microsporus	*Endornaviridae*	*Alphaendornavirus*	Rhizopus microsporus endornavirus 1	ssRNA(+)	LC671616
15	R. microsporus	*Endornaviridae*	*Alphaendornavirus*	Rhizopus microsporus endornavirus 2	ssRNA(+)	LC671617
15	R. microsporus	*Mitoviridae*	Unclassified	Rhizopus microsporus mitovirus 1	ssRNA(+)	LC671615
56	Cladosporium sphaerospermum	*Botourmiaviridae*	*Penoulivirus*	Erysiphe necator associated ourmia-like virus 69	ssRNA(+)	LC671618
60	P. vanoranjei	Unclassified	Unclassified	Penicillium vanoranjei associated RNA virus 1	RNA	LC671619
61	A. lentulus	*Botourmiaviridae*	*Magoulivirus*	Aspergillus fumigatus botourmiavirus 1	ssRNA(+)	LC671624
61	A. lentulus	*Narnaviridae*	Unclassified	Aspergillus fumigatus narnavirus 2	ssRNA(+)	LC671623
61	A. lentulus	Unclassified	Unclassified	Aspergillus lentulus jivivirus 1	RNA	LC671620 LC671621 LC671622

### DNA virus discovery in RCA libraries.

Analysis of VIDISCA sequencing reads identified no DNA viruses in the 61 analyzed samples, and we therefore focused instead on the Illumina libraries enriched by rolling circle amplification (RCA) for circular ssDNA. These had an average of 1.7 million reads per library after quality control. Removal of poor quality contigs left 14 that appeared to be of cressdnaviral origin, since they possessed at least partial Rep and Cap proteins with BLASTp identity to known viruses. We found that 12 of the 14 sequences were complete circular genomes and the last two were truncated. Each was derived from a different sample. Since RCA indiscriminately amplifies any primed circular ssDNA, we explored the possibility that they represented viral contaminants amplified from reagents rather than mycoviruses (26, 27). We first looked at the distribution of reads mapping to each sequence across all RCA libraries. We found that one of the incomplete genomes had a clear signature of contamination, being positive in 11 of 61 samples at a cutoff of 50 reads per million (RPM, 18% prevalence), with 39 samples containing at least one read (Table S4). On closer examination, this sequence was also found to contain a region with BLASTn identity to the fungal isolate species, suggesting a hybrid assembly. Despite this, after trimming off the hybrid region, the contaminant mapping signature remained. We opted to retain the Rep sequence for phylogenetic analysis, but did not upload the nucleotide sequence to the International Nucleotide Sequence Database Collaboration (INSDC) databases due to its uncertain quality (instead providing it at https://figshare.com/projects/Viruses_infecting_clinical_mycology_cultures/128186). The other 13 sequences (Table 2) did not contain hybrid regions, and also showed more specific distributions, being positive in between one and three samples (1.6% to 4.9% prevalence). This aligned more with our expectations of mycoviruses rather than contaminants. Despite this, PCR screening failed to detect the 13 sequences in unamplified index samples, while off-target amplification showed the polymerase was active. We hypothesized that this could be explained by low viral load combined with poor PCR efficiency, and so repeated the RCA step with freshly extracted nucleic acids. PCR on the amplified nucleic acids again failed to detect the viruses, suggesting they represent low-level contamination of a reagent or reagents used upstream of RCA. This result shows the importance of validation assays, and underscores that contaminants will not necessarily be widely distributed across samples, presumably due to a low initial load and related sampling effects. None of the 13 sequences dominated their respective libraries, with 3,869 RPM the maximum normalized read count, in line with low load prior to RCA. Previous work in our laboratory on the circular anelloviruses has shown RCA can amplify genomes to a level allowing complete assembly, even from loads below PCR and qPCR limits of detection (28). Together, the result strongly implies caution is needed in interpreting the biological source of amplified viruses, as incorrect host predictions could easily occur.

**TABLE 2:** Contaminant circular single-stranded DNA viruses in RCA libraries constructed from clinical isolates of fungi

Sample	Virus order	Virus name	Molecule	Accession
1	*Arfiviricetes*	*Cressdnaviricota* sp. isolate 2020-AMS-AF	ssDNA	LC671629
3	*Arfiviricetes*	*Cressdnaviricota* sp. isolate 2020-AMS-AN	ssDNA	LC671630
12	*Arfiviricetes*	*Cressdnaviricota* sp. isolate 2020-AMS-CB	ssDNA	LC671631
17	*Arfiviricetes*	*Cressdnaviricota* sp. isolate 2020-AMS-MCA	ssDNA	LC671632
19	*Arfiviricetes*	*Cressdnaviricota* sp. isolate 2020-AMS-MP	ssDNA	LC671633
29	*Arfiviricetes*	*Cressdnaviricota* sp. isolate 2020-AMS-MCO	ssDNA	LC671634
31	*Arfiviricetes*	*Cressdnaviricota* sp. isolate 2020-AMS-TR	ssDNA	LC671625
33	*Arfiviricetes*	*Cressdnaviricota* sp. isolate 2020-AMS-TS	ssDNA	LC671626
40	*Arfiviricetes*	*Cressdnaviricota* sp. isolate 2020-AMS-SB	ssDNA	LC671627
42	*Arfiviricetes*	*Cressdnaviricota* sp. isolate 2020-AMS-ED	ssDNA	LC671637
46	*Arfiviricetes*	*Cressdnaviricota* sp. isolate 2020-AMS-AK	ssDNA	LC671636
49	*Arfiviricetes*	*Cressdnaviricota* sp. isolate 2020-AMS-RA	ssDNA	LC671628
54	*Arfiviricetes*	*Cressdnaviricota* sp. isolate 2020-AMS-SP	ssDNA	LC671635

### Virus relationships and taxonomy.

**FIG 2:**
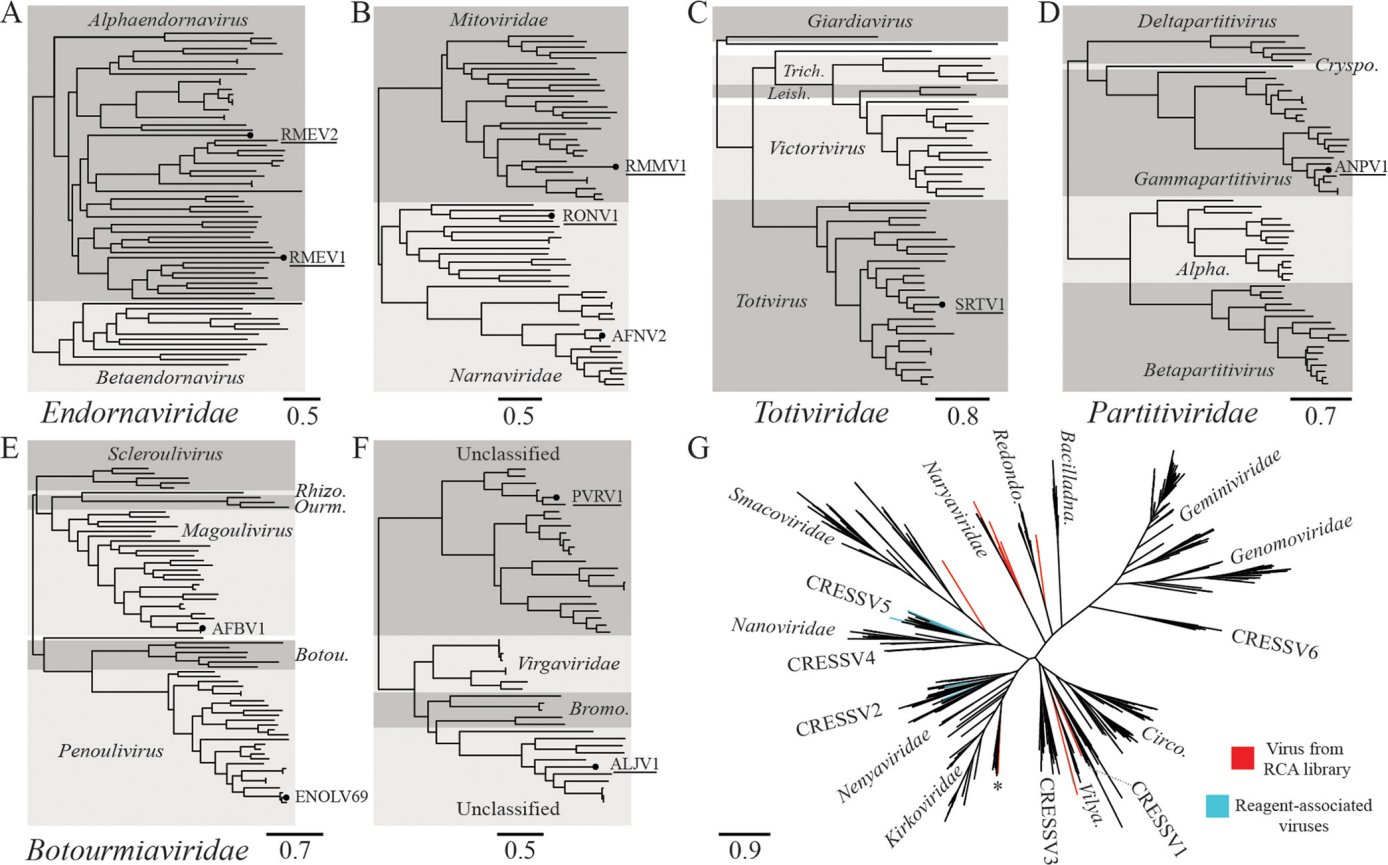
Phylogenetic relationships of viruses. Scale bars refer to amino acid substitutions per site. An underlined virus name denotes a viral genome likely meeting criteria for a new species. (A) *Endornaviridae*, Rhizopus microsporus endornavirus 1 (RMEV1, LC671616), Rhizopus microsporus endornavirus 2 (RMEV2, LC671617). (B) *Mitoviridae* and *Narnaviridae*, Rhizopus microsporus mitovirus 1 (RMMV1, LC671615), Rhizopus oryzae narnavirus 1 (RONV1, LC671613), Aspergillus fumigatus narnavirus 2 (AFNV2, LC671623, isolated here from Aspergillus lentulus). (C) *Totiviridae*, Syncephalastrum racemosum totivirus 1 (SRTV1, LC671614); *Trich*., *Trichomonasvirus*; *Leish*., *Leishmaniavirus*. (D) *Partitiviridae*, Aspergillus niger partitivirus 1 (ANPV1, LC671611); *Cryspo.*, *Cryspovirus*; *Alpha.*, *Alphapartitivirus*. (E) *Botourmiaviridae*, Aspergillus fumigatus botourmiavirus 1 (AFBV1, LC671624, isolated here from Aspergillus lentulus), Erysiphe necator-associated ourmia-like virus 69 (ENOLV69, LC671618, isolated here from *Cladosporium sphaerospermum*); *Rhizo.*, *Rhizoulivirus*; *Ourm*., *Ourmiavirus*; *Botou*., *Botoulivirus*. (F) Unclassified RNA viruses: Penicillium vanoranjei-associated RNA virus 1 (PVRV1, LC671619); Aspergillus lentulus jivivirus 1 (ALJV1, LC671620); *Bromo*., *Bromoviridae*. (G) *Cressdnaviricota*: 14 Rep sequences from viruses found in RCA libraries are highlighted in red, while known reagent-associated viruses are highlighted in blue. Asterisked sequence was a hybrid assembly and therefore not uploaded to INSDC databases. *Bacilladna.*, *Bacilladnaviridae*; *Redondo.*, *Redondoviridae*; *Vilya.*, *Vilyaviridae*; *Circo.*, *Circoviridae*. Alignments, tree files, and the hybrid sequence are available at https://figshare.com/projects/Viruses_infecting_clinical_mycology_cultures/128186.

Two members of the family *Endornaviridae* were found in a single Rhizopus microsporus culture. They were distantly related to each other, sharing 13% amino acid (aa) identity across the RdRp, and interestingly both were distant to all other public endornavirus sequences. The Committee on Taxonomy of Viruses (ICTV) species demarcation for endornaviruses is <75% nucleotide (nt) identity across the genome (29), and both sequences would qualify as new species by this criterion. As the first endornaviruses identified in R. microsporus, we tentatively named them Rhizopus microsporus endornavirus 1 and 2 (RMEV1 and RMEV2). The closest relative of RMEV1 was Rhizoctonia solani endornavirus 7 (QDW65434.1) at 15.6% RdRp aa identity, while for RMEV2 it was Phytophthora endornavirus 2 (BCL84886.1) at 17.14% RdRp aa identity. Phylogenetic analysis showed both RMEV1 and RMEV2 cluster within the genus *Alphaendornavirus* (Fig. 2A), consistent with their relatively large genomes of 13,589 bp and 11,599 bp, respectively. Alphaendornaviruses are currently known to infect fungi, plants, and oomycetes, and hypovirulent effects on hosts have been observed in some cases, for example the alphaendornavirus Helicobasidium mompa endornavirus 1 (30).

One member of the family *Mitoviridae* and two members of the family *Narnaviridae* were identified. The mitovirus coinfected the same R. microsporus culture as RMEV1 and RMEV2, though while endornavirus replication is cytoplasmic, mitoviruses replicate in fungal mitochondria (31). Mitovirus genus and species demarcation criteria have yet to be defined, but <40% RdRp aa identity has historically been found between defined species (32). On this basis, we suggest the genome be named Rhizopus microsporus mitovirus 1 (RMMV1), with Entomophthora muscae mitovirus 2 (QCF24461.1) as the closest relative (37.4% RdRp aa identity). Narnaviruses were found in cultures of Rhizopus oryzae and Aspergillus lentulus. They shared only 8% RdRp aa with each other and clustered in different parts of the family tree (Fig. 2B). Genus demarcation for narnaviruses has not been defined, but the species cutoff is <50% RdRp aa identity (32). The former genome met this criterion, and we suggest the name Rhizopus oryzae narnavirus 1 (RONV1) for it, which has 36.6% RdRp aa identity to its closest relative, Erysiphe necator-associated narnavirus 42 (QJT93774.1). The virus found in A. lentulus belongs to the previously described species Aspergillus fumigatus narnavirus 2 (AFNV2), sharing 98% RdRp aa identity with accession AXE72934.1. Although no data on biological impact are currently available for these viruses, some mitoviruses and narnaviruses do have the potential to impact fungal biology either by conferring hypovirulence or affecting reproductive capabilities (33, 34).

A member of the family *Totiviridae* was found in a Syncephalastrum racemosum culture. Phylogenetic analysis placed it within the genus *Totivirus* (Fig. 2C). Species demarcation criteria for the *Totivirus* genus are not absolute, and largely relate to biological characteristics such as host range (though <50% RdRp aa identity is also considered a probable species cutoff). The closest relative of the virus identified here was Trichoderma koningiopsis totivirus 1 (QGA70771.1) at 60.9% RdRp aa identity. Despite this, we suggest the genome be given the provisional name Syncephalastrum racemosum totivirus 1 (SRTV1). Our rationale is the large phylogenetic distance between the host genera *Syncephalastrum* and *Trichoderma* and the current lack of biological data to support assignment to the same species. Members of the genus *Totivirus* have been previously associated with hypovirulence (35).

A virus belonging to the family *Partitiviridae* was identified in a culture of Aspergillus niger. The sequence was phylogenetically placed within the genus *Gammapartitivirus* (Fig. 2D). Criteria for species demarcation within this genus are <90% RdRp aa identity and also <80% capsid aa identity (36), and the sequence identified here meets this, most closely related to Botryosphaeria dothidea virus 1 (KJ722537.1) with 77.1% RdRp aa identity and 54.9% capsid aa identity. We suggest the name Aspergillus niger partitivirus 1 (ANPV1). The finding is in line with the known ascomycete host range of the genus *Gammapartitivirus.*

Two members of the family *Botourmiaviridae* were found, one in the A. lentulus culture also containing AFNV2 and another in a *Cladosporium sphaerospermum* culture. The former belonged to the genus *Magoulivirus*, while the latter belonged to the genus *Penoulivirus* (Fig. 2E). The species demarcation criterion for both these genera is <90% RdRp aa identity, and neither met this; the magoulivirus belongs to Aspergillus fumigatus botourmiavirus 1 (AFBV1, BCH36640.1) with 97.7% RdRp aa identity, while the penoulivirus belongs to Erysiphe necator-associated ourmia-like virus 69 (QKI79899.1) with 90.7% RdRp aa identity. At least one member of the family has previously been shown to be associated with the hypovirulence of its host (37).

The final two RNA viruses identified were both unclassified. One coinfected the A. lentulus culture alongside AFNV2 and AFBV1, while the other was found in a *Penicillium vanoranjei* culture. BLASTp searches showed the closest relatives of the A. lentulus virus included Citrus virga-like virus (CVLV, ARO38274.1) and Grapevine-associated jivivirus 1 (QIJ25698.1), each with approximately 40% RdRp aa identity across >96% query coverage. Though no ICTV guidelines on species demarcation currently exist for this lineage, we propose this genome be named Aspergillus lentulus jivivirus 1 (ALJV1) on the basis of low sequence identity to relatives (39% RdRp aa identity across the whole protein alignment to CVLV) in combination with a novel host record; notably, this is the first jivivirus identification in an axenic fungal culture. As the first record, no data are currently available regarding the biological impact of jivivirus infection on their fungal hosts. The closest relative of the virus infecting *P. vanoranjei* was an unclassified virus recorded as *Riboviria* sp. (QDH88072.1), at 49% RdRp aa identity. We gave it the temporary name Penicillium vanoranjei-associated RNA virus 1 (PVRV1) until proper taxonomic classification. Notably, BLASTp results suggested ALJV1 was related to the *Virgaviridae* and *Bromoviridae*, while PVRV1 hit one sequence labeled as virga-like (BBB86779.1). We therefore analyzed their relationships together, alongside representatives of both families. This confirmed a close relationship between ALJV1 and members of the *Virgaviridae* and *Bromoviridae* (Fig. 2F). PVRV1 was resolved in a distinct lineage alongside other unclassified viruses, many of which were themselves found associated with fungi.

As described above, analysis of RCA libraries returned 14 cressdnavirus sequences suspected of being contaminants due to PCR validation failure in the original samples. Phylogenetic analysis of the Rep proteins showed they clustered in distinct locations across the *Arfiviricetes* class (Fig. 2G). The only family of cressdnaviruses currently recognized to infect fungi are the family *Genomoviridae*, belonging to the class *Repensiviricetes*. This is concordant with a nonfungal host of these viruses. Largely, the 14 Rep sequences could not be assigned to recognized clusters or families (except one apparent member of *Naryaviridae*), but all remaining sequences were resolved as distant relatives of lineages, including *Kirkoviridae*, *Smacoviridae*, *Naryaviridae*, *Redondoviridae*, CRESSV1, and *Vilyaviridae*. These lineages in particular are conspicuous since all are found associated with the gastrointestinal tracts of humans and other animals, and also in the human respiratory environment in the case of *Redondoviridae* (38). Gastrointestinal viruses are often detected in stool-contaminated wastewater. While unconfirmed, if the viruses detected here occupy similar niches, it may suggest the true contamination source is recycled water. The 14 viral Reps were mostly unrelated to sequences previously identified as contaminants (26), though one (LC671626) did cluster alongside MZ824233.1 and MZ824234.1.

## DISCUSSION

Decades of research have uncovered numerous mycoviruses, with the bulk of sampling effort directed toward industrially relevant hosts, such as plant-pathogenic fungi or edible mushrooms (39, 40). Large-scale efforts to genetically catalogue mycoviruses of human-pathogenic fungi specifically have been limited (40), though there has long been evidence they also host viruses (39), and genomes are now increasingly becoming available on public databases. Here, we investigated the viruses of 75 clinical isolates of medically relevant fungi, covering 27 genera and at least 56 species. We uncovered a remarkable diversity of 11 RNA mycovirus genomes in seven hosts. This probably represents an underestimate of the true RNA virus richness in our sample set, since even within the seven deep-sequenced samples, we detected five viruses not observed with initial VIDISCA screening. While we currently lack data on the biological impact of these viruses, many are related to viruses capable of conferring hypovirulence on their hosts. Despite this, the majority of mycoviruses do not negatively impact their hosts (41), and each must be individually characterized, for example by comparing growth characteristics of infected cultures with virus-free ones. A notable possibility is that clinically isolated fungi may be particularly poor sources for discovery of hypovirulence-associated viruses, since they are competent pathogens upon isolation. Screening of fungi in their alternative niches might therefore be more productive in this regard. Aside from hypovirulence, the therapeutic potential of RNA mycoviruses is generally unfavourable, since all studied to date lack an extracellular stage, possibly due to a physical inability to transit pores in fungal cell walls (25). Discovery of smaller ssDNA viruses may circumvent this barrier.

Detected mycoviruses belonged to six families and two additional unclassified groups. Only in two cases were their closest known relatives also identified in a human-pathogenic fungus (AFBV1 and AFNV2, from Aspergillus fumigatus). In five cases, the closest mycovirus relatives were identified in phytopathogenic fungi, one was found in an endophytic species, one in an entomopathogenic fungus, and in two cases the relatives were from uncertain hosts. This is likely partly due to low sampling effort toward human pathogens as mentioned above; however, it probably also reflects the fact that human-pathogenic fungi are phylogenetically nested within nonpathogenic lineages across the fungal radiation (42) and consequently share their viral lineages. Indeed, human-pathogenic fungi are mostly opportunistic rather than obligate pathogens (42), normally filling other ecological roles where mycovirus host switches could occur. For example, Rhizopus microsporus is both human- and plant-pathogenic, and its virome might therefore be expected to resemble other phytopathogens.

This study focused on fungi recultured from axenic stocks, with all RNA viruses confirmed by PCR in their original sample extractions. The viral phylogenetic relationships were also concordant with previous mycovirus literature or public sequences (except ALJV1, see below), and we were thus confident they represented true mycoviruses and not contamination. The notable exception was ALJV1, which represents the first unambiguous detection of a jivivirus in a fungus. Previous identifications of the recently named jiviviruses (43) have been plant or plant-pest associated (thrips), though they have also been observed associated with *Plasmopara-*infected grapevines (43). Besides fungi, it is therefore probable they infect plants, and potentially oomycetes. The unclassified lineage containing jiviviruses is related to the families *Virgaviridae* and *Bromoviridae*, both of which infect plants (44, 45). Interestingly, virga-like viruses are also known from a fungus (46), and cucumber mosaic virus (CMV, family *Bromoviridae*) has been observed to naturally infect the phytopathogenic fungus Rhizoctonia solani, which can in turn transmit CMV to uninfected plants under laboratory conditions (47). Such cross-kingdom transmission may similarly occur with jiviviruses. Interestingly, most lineages of mycoviruses have plant virus relatives (48), hinting at a deep history of cross-kingdom host shifts during extensive ecological interaction. This is true for most families identified here; indeed, some members of the *Endornaviridae*, *Botourmiaviridae*, *Mitoviridae*, *Partitiviridae*, and *Totiviridae* can all infect plants (48). Cross-kingdom transmission has been suggested to have played a major role in the evolution of mycoviruses (49).

We also detected ssDNA viruses in RCA libraries, 12 with complete genomes. We universally failed to validate these by PCR, both in the original unamplified samples and after repeating RCA. Recently, more attention has been given to the detection of viral contaminants in sequencing libraries, since they can easily result in incorrect assessments of virus–host relationships (26, 50). Here, we found that contaminating sequences can occur in RCA libraries without a wide distribution as may be expected, but rather occurring in between one and three samples each. This serves as a further caution that validation assays are essential to confirm the presence of viruses in samples. Our failure to detect DNA viruses is perhaps unsurprising, given their relative rarity among mycoviruses (13). Despite finding no ssDNA mycoviruses here, we reiterate the rationale that discovering hypovirulence-associated ssDNA viruses with an extracellular stage may represent the best opportunities for application in human therapeutics, and we therefore suggest RCA should still be applied in similar surveys of human pathogens.

## MATERIALS AND METHODS

### Fungal clinical isolates.

Bronchial aspirates, bronchoalveolar lavage fluid, bone marrow, and biopsy specimens sent in for culture of (dimorphic) fungi were inoculated on two containers of brain heart infusion agar with penicillin and gentamicin. One container was incubated for 3 weeks at 20 to 25°C, and the other at 35 to 37°C. Nails, hair, and skin scrapings were inoculated on dermatophyte test medium agar and Sabouraud agar with gentamicin and chloramphenicol (SabGC). Incubation was for 3 weeks at 25 to 28°C, with one container of SabGC incubated for 3 weeks at 35 to 37°C. All other materials sent in for fungal culture were inoculated on two SabGC containers for 1 week, one at 25 to 28°C and one at 35 to 37°C. Fungal isolates included in this study were recultured from glycerol stocks, and samples were transferred to tubes containing Universal Transport Medium (UTM, Copan). A total of 75 isolates were included, split into two batches (Table S1). Batch one samples (61 diverse isolates) were utilized in virus discovery and PCR screening experiments, while batch two samples (14 isolates of *Mucorales* species) were included later and used only in PCR screening.

### Next generation sequencing.

Fungal swabs were suspended 1:3 in UTM. Sample suspension (110 μL) was transferred to a reaction tube and centrifuged (10 min at 5,000 *g*) to pellet solid matter and cellular debris. Supernatant was treated with 20 μL TURBO DNase (Thermo Fisher Scientific, Waltham, MA, USA) for 30 min at 37°C to remove naked DNA. Nucleic acids were extracted using the Boom method (51) and were then split according to their use either in VIDISCA or RCA library preparation. For VIDISCA, reverse transcription (RT) was done on 20 μL using nonribosomal hexamer primers (52). This was followed by second-strand synthesis and a cleanup via phenol-chloroform extraction and ethanol precipitation. Double-stranded DNA was digested with MseI restriction enzyme, and sequencing adapters were ligated to the sticky ends. Libraries were amplified before size selection of fragments between 200 and 600 bp, quantification, and pooling. Sequencing was then done on an IonTorrent S5 instrument. For RCA, 4 μL of extracted nucleic acids was incubated with Φ29 DNA polymerase and exonuclease-resistant random primers for 4 h at 30°C. Product was incubated with NEBNext dsDNA fragmentase (New England Biolabs) for 25 min at 37°C and then cleaned up, which was also done after each subsequent step. Fragmented DNA was end repaired for 30 min at 37°C using the Klenow fragment of DNA polymerase I (New England Biolabs) before A-tailing was carried out for 30 min at 37°C with Klenow fragment (3′→5′ exo-, New England Biolabs). NEBNext adapters (1:1,000 dilution, New England Biolabs) were ligated overnight at 16°C using T4 DNA ligase (5 U/μL, Invitrogen). After size selection of fragments >200 bp, adaptor-ligated DNA was treated with USER enzyme (New England Biolabs) and was then enriched and indexed during a 12-cycle PCR. Further size selection to target fragments between 200 and 600 bp was done, before quantification, pooling, and paired-end sequencing (2 × 150 bp) on an Illumina MiSeq instrument. Total nucleic acid metagenomic sequencing was carried out on samples positive for RNA viruses after VIDISCA sequencing. Library preparation up to second-strand synthesis was identical to the VIDISCA protocol described above, except that RT hexamers carried a 5′-phosphate and AMPure XP beads were used for all cleanups. After second-strand synthesis, the protocol matched RCA methodology from fragmentation onward. The library preparation and sequencing was carried out twice independently. Raw Illumina reads are available under European Nucleotide Archive project accession PRJEB49942.

### Virus discovery and genome assembly.

VIDISCA sequences were analyzed with a previously published workflow (53). Briefly, reads were aligned to viral proteins using the UBLAST algorithm (54), before reduction of false positives by alignment of hits to the GenBank nt database using BLASTn (55). Visual outputs generated from hit tables were inspected to identify viral content, and samples positive for RNA viruses were selected for RNA deep sequencing. Illumina sequence reads from virus-enriched metagenomic libraries and RCA products were cleaned of adapters and quality trimmed to a Phred score of 30 using BBDuk, from BBMap v38.71. *De novo* assembly was done with SPAdes v3.15.2 (56). Contigs from the metagenomic libraries were aligned to a database of viral proteins using UBLAST to identify putative RNA viruses. Contigs generated from RCA products were aligned to a database of Rep genes covering *Cressdnaviricota* diversity. Matching contigs above 1,500 bp were aligned to the GenBank nt database using BLASTn to remove nonviral sequences. Remaining contigs were self-aligned using the MAFFT online server (https://mafft.cbrc.jp/alignment/server/), and those containing visible misassemblies were discarded. Genome completeness was assessed by the presence of both *Rep* and *Cap* genes plus genome circularity (identical sequence at both contig ends). Circular overlap was trimmed from complete genomes, and they were rotated to begin with the *Rep* gene. For all RNA and DNA genomes, inspection was performed by mapping quality controlled reads to respective sequences using BWA MEM v0.7.17 (57) and manually examining resulting pileups. Contigs were curated to correct minor errors, though assemblies with uncorrectable misassemblies were discarded.

### PCR validation and analysis of viral distribution.

To confirm viral RNA in index samples, and assess distribution across the others, PCRs were designed and run on freshly extracted and reverse transcribed nucleic acids from all samples (for primers, see Table S2). A 40-cycle first round was performed, with nested PCR carried out if this was negative, and PCR products were Sanger sequenced. For ssDNA viruses, only index samples were screened due to nondetection. Both the original unamplified samples and an aliquot with RCA repeated were screened. To explore the possibility that they represented contaminants of reagents, we also examined ssDNA virus read distribution across all RCA libraries. Since reagents were shared between samples, we expected that contaminants would be detected in all or most sequencing libraries. This was tested by mapping reads from all RCA libraries to the set of cressdnaviral sequences using BWA and examining their read distributions per sample, using 50 reads per million (RPM) as the cutoff for positive detection.

### Phylogenetic and genetic distance analyses.

For each RNA virus group, representative RNA-dependent RNA polymerase (RdRp) or polyprotein sequences were gathered using the International Committee on Taxonomy of Viruses (ICTV) website and GenBank, and alignment was performed using MAFFT v7.490 (58) with the E-INS-i setting. Open reading frame prediction for genomic segments was done using default ORFfinder settings (https://www.ncbi.nlm.nih.gov/orffinder), though the yeast mitochondrial genetic code was applied for Rhizopus microsporus mitovirus 1 (LC671615), since its UGA codon encodes tryptophan rather than a translation termination signal (31). Maximum likelihood phylogenetic analyses were done using IQ-TREE v1.6.11 (59) with automatic model detection, 1,000 ultrafast bootstrap tests, and 1,000 SH-aLRT tests. Pairwise distances between proteins were calculated for each alignment, using the SIAS web tool with mean length of sequences set as the denominator (http://imed.med.ucm.es/Tools/sias.html). For cressdnaviruses, Rep protein sequences were aligned alongside a database covering recognized and proposed lineages collated for a previously published phylogeny (60), now with the addition of the proposed family *Kirkoviridae* (61) and seven Rep sequences previously shown to be reagent associated (26). Phylogenetic analysis was as above. All alignments and tree files are available at https://figshare.com/projects/Viruses_infecting_clinical_mycology_cultures/128186.

### Data availability.

Assembled genomes are available from INSDC databases under accessions LC671611–LC671637. Raw Illumina reads are available under European Nucleotide Archive project accession PRJEB49942. Alignments, tree files, and one hybrid sequence are available from https://figshare.com/projects/Viruses_infecting_clinical_mycology_cultures/128186.
